# Deciphering the Glycolipid Code of Alzheimer's and Parkinson's Amyloid Proteins Allowed the Creation of a Universal Ganglioside-Binding Peptide

**DOI:** 10.1371/journal.pone.0104751

**Published:** 2014-08-20

**Authors:** Nouara Yahi, Jacques Fantini

**Affiliations:** Aix-Marseille Université, PPSN-EA4674, Faculté des Sciences, Marseille, France; University of South Florida College of Medicine, United States of America

## Abstract

A broad range of microbial and amyloid proteins interact with cell surface glycolipids which behave as infectivity and/or toxicity cofactors in human pathologies. Here we have deciphered the biochemical code that determines the glycolipid-binding specificity of two major amyloid proteins, Alzheimer's β-amyloid peptide (Aβ) and Parkinson's disease associated protein α-synuclein. We showed that both proteins interact with selected glycolipids through a common loop-shaped motif exhibiting little sequence homology. This 12-residue domain corresponded to fragments 34-45 of α-synuclein and 5-16 of Aβ. By modulating the amino acid sequence of α-synuclein at only two positions in which we introduced a pair of histidine residues found in Aβ, we created a chimeric α-synuclein/Aβ peptide with extended ganglioside-binding properties. This chimeric peptide retained the property of α-synuclein to recognize GM3, and acquired the capacity to recognize GM1 (an Aβ-inherited characteristic). Free histidine (but not tryptophan or asparagine) and Zn^2+^ (but not Na^+^) prevented this interaction, confirming the key role of His-13 and His-14 in ganglioside binding. Molecular dynamics studies suggested that the chimeric peptide recognized cholesterol-constrained conformers of GM1, including typical chalice-shaped dimers, that are representative of the condensed cholesterol-ganglioside complexes found in lipid raft domains of the plasma membrane of neural cells. Correspondingly, the peptide had a particular affinity for raft-like membranes containing both GM1 and cholesterol. The chimeric peptide also interacted with several other gangliosides, including major brain gangliosides (GM4, GD1a, GD1b, and GT1b) but not with neutral glycolipids such as GlcCer, LacCer or asialo-GM1. It could inhibit the binding of Aβ1-42 onto neural SH-SY5Y cells and did not induce toxicity in these cells. In conclusion, deciphering the glycolipid code of amyloid proteins allowed us to create a universal ganglioside-binding peptide of only 12-residues with potential therapeutic applications in infectious and neurodegenerative diseases that involve cell surface gangliosides as receptors.

## Introduction

Plasma membrane glycolipids serve as primary attachment sites for a broad range of infectious and amyloid proteins [1]. Thus, understanding how these proteins physically interact with glycolipids is of high interest. Because only the glycone part of glycolipids is directly accessible to extracellular proteins, molecular evolution has allowed the emergence of a specific protein domain compatible with the sugar head groups of cell surface glycolipids [2]. This domain, discovered in 2002 in viral and amyloidogenic proteins, has been referred to as a universal sphingolipid-binding domain (SBD) [3]. The SBD is defined as a structurally conserved loop motif exhibiting little amino acid sequence homology [4]. It has been found in numerous cellular [5]–[8] and pathogen proteins [9]–[12] that recognize sphingomyelin, and/or glycophingolipids. In a recent study focused on the glycolipid binding specificity of α-synuclein, the protein associated with Parkinson's disease, we have identified the 34-45 hairpin segment as the shortest active glycolipid-binding domain that displays the glycolipid-binding properties of the whole α-synuclein [13]. Indeed, this short linear motif of 12 amino acid residues confers to the protein a high specificity of interaction for GM3 [13], [14], a ganglioside abundantly expressed by astrocytes [15]. The 34-45 domain of α-synuclein shares structural homology with the 5–16 fragment of Alzheimer's β-amyloid peptide (Aβ), yet the glycolipid-binding domain of Aβ does not recognize GM3, but GM1. In this case, the ganglioside-binding specificity of these amyloid proteins may lead to a preferential interaction with distinct cellular targets, e.g. GM3-expressing astrocytes [15] for α-synuclein, and GM1-enriched post-synaptic membranes [16] for Aβ. In the human brain, the expression of GM1 gradually increases with age, whereas in parallel GM3 content diminishes [17]. Both GM1 and GM3 have been involved in the pathophysiology of Alzheimer's and Parkinson's diseases [18]–[20]. For all these reasons, it is of high interest to understand how amyloid proteins interact with brain glycolipids. This is not an easy task because the same SBD can mediate the interactions with various glycolipids, as reported for both Aβ and α-synuclein [13]. Conversely, the same glycolipid can be recognized by the respective SBD of phylogenetically-distant proteins, as it is the case for GM3, a ganglioside recognized by HIV-1 gp120 [21], human α-synuclein, [13] and the cellular prion protein PrP [22]. Recently, we demonstrated that each SBD contains a subset of key amino acid residues that are physically involved in glycolipid binding [13]. Identifying these residues and understanding why they confer a particular specificity for a given SBD/glycolipid couple is a critical step to elucidate the molecular mechanisms controling protein/glycolipid interactions. In this respect, we have recently proposed that these interactions are governed by a biochemical code and that a rational strategy to decipher this code is to study protein/glycolipid interactions with minimal synthetic SBD peptides [13]. In the present study we have used both wild-type and mutant peptides derived from the respective SBDs of Aβ and α-synuclein. Through a combination of in silico, physico-chemical, and cellular approaches, we have dissected the molecular mechanisms accounting for the ganglioside-binding specificity of Aβ and α-synuclein. These results have enabled us to create a chimeric α-synuclein/Aβ peptide displaying the ganglioside-binding properties of both proteins.

## Materials and Methods

### Materials

Synthetic peptides with a purity >95% were obtained from Schafer-N (Copenhagen, Denmark). Ultrapure apyrogenic water was from Biorad (Marnes La Coquette, France). All lipids were purchased from Matreya (Pleasant Gap, PA). Biotin-conjugated Aβ1-42 with a 6-carbon long chain (LC) was from AnaSpec (Fremont, CA). The α-syn/HH peptide has been patented under the number 14305353.6 - 1408.

### Molecular modeling

In silico studies of peptide-ganglioside interactions were performed with the Hyperchem 8 program (ChemCAD, Obernay, France) as described previously [13], [14]. Briefly, geometry optimization of ganglioside dimers and ganglioside-peptide complexes was achieved using the unconstrained optimization rendered by the Polak-Ribière conjugate gradient algorithm. Molecular dynamics simulations were performed for iterative periods of times of 1 ns in vacuo with the Bio+ (CHARMM) force field [23]. The molecules were visualized with Hyperchem 8, PDB-viewer [24] and Molegro Molecular Viewer [25] softwares. The energies of interaction were estimated with the ligand energy inspector function of Molegro Molecular Viewer [25].

*Lipid monolayer assay*. Peptide-cholesterol interactions were studied with the Langmuir film balance technique [26] using a Kibron Inc. (Helsinki, Finland) microtensiometer as previously described [13], [27]. Monomolecular films of pure lipids (or lipid mixtures) were spread on the indicated subphase (pure water, presence of salt or amino acids when indicated). After spreading of the film, 2 min was allowed for solvent evaporation. The peptide (or the protein) was injected in the subphase (pH 7) with a 10-µl Hamilton syringe, and the surface pressure increases produced by the peptide were continuously recorded as a function of time. The data were analyzed with the FilmWareX program (Kibron Inc.).

### Aβ1-42 binding assay

SH-SY5Y cells were seeded in 96-well plates at a density of 30.000 cells per well. After three days, the cells were rinsed in phosphate buffered saline (PBS-Ca^2+^), and fixed with 3.7% paraformaldehyde for 10 min at room temperature. All subsequent steps were performed at room temperature. After rinsing in PBS-Ca^2+^, the cells were saturated for 1hr with lipid-free bovine serum albumin (2%), then incubated for 2 hr with biotin-labeled Aβ1-42 [28] at a concentration of 8 µg.mL^-1^, in absence or presence of various concentrations of the chimeric α-syn34-45 peptide. The cells were then rinsed and incubated with Streptavidin-HRP (Sigma, St Louis, MO) for 1 hr. Sigma-Fast OPD (Sigma) was used as revealing agent. The reaction was stopped with H_2_SO_4_ 2N and the absorbance was measured at 492 nm. Specific Aβ1-42 binding was estimated against blank experiments (absorbance measured on cells incubated with Streptavidin-HRP alone and revealed with Sigma-Fast OPD).

### Cell viability assay

Cell viability was assed using the 3-(4,5-dimethylthiazol-2-yl)-5-(3-carboxymethoxyphenyl)-2-(4-sulfophenyl)-2H-tetrazolium (MTS) assay (Promega, Madison, WI). After peptide treatment of SH-SY5Y cells in 96-well plates (24hr in serum-free medium), MTS was added (20 µL/100 µL) and the cells were incubated during 3 hr at 37°C. MTS was spectrophotometrically measured at 490nm.

## Results

### Role of histidine residues in Aβ binding to ganglioside GM1

**Figure 1:**
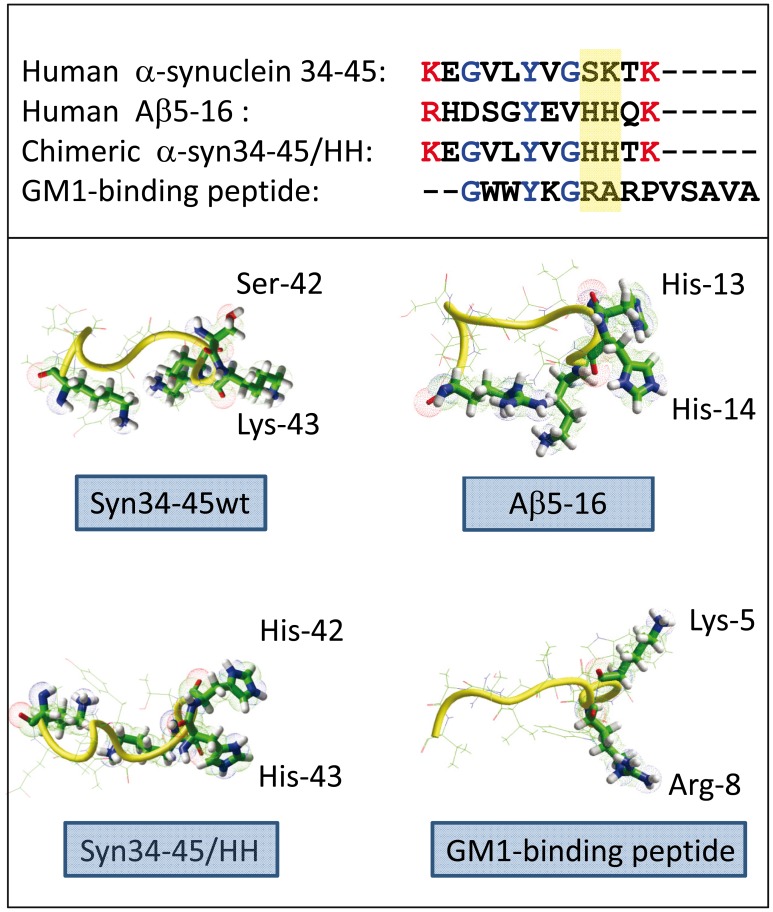
Amino acid sequence and structure of the common glycolipid-binding domains of Aβ, α-synuclein, and of the chimeric α-syn34-45/HH peptide. The amino acid sequences of the peptides used in the present study are indicated in the upper panel. For comparison, the sequence of a GM1-binding peptide identified through a phage display selection strategy is also shown. The positions 42–43 (α-synuclein), 13–14 (Aβ), and 7–8 (GM1-binding peptide) are framed in yellow. The minimized 3D structures of these peptides in vacuo are shown in the lower panel.

**Figure 2:**
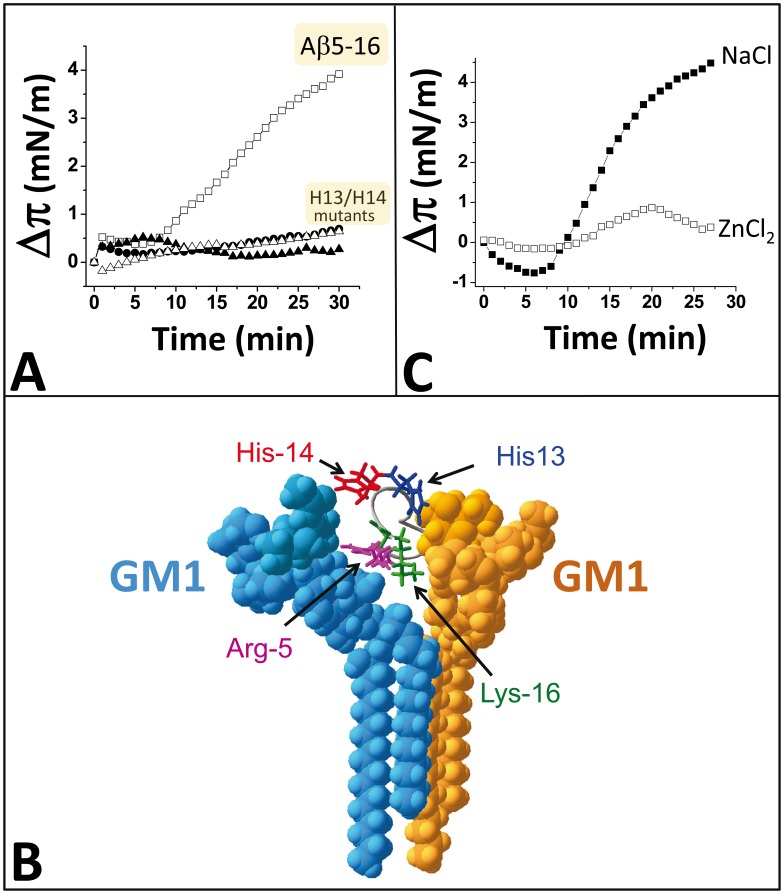
Both His-13 and His-14 residues are involved in the binding of Aβ5-16 to GM1. **A.** A monolayer of ganglioside GM1 was prepared at an initial surface pressure of 17.5 mN.m^-1^. After equilibration, the wild-type Aβ5-16 (open squares), or mutant Aβ5-16/H13A (full triangles), Aβ5-16/H14A (full circles), Aβ5-16/H13A/H14A (open triangles) peptides were injected in the aqueous subphase underneath the monolayer. The data show the evolution of the surface pressure π following the injection of peptides (10 µM) in the aqueous subphase underneath the monolayer. Each experiment was performed in triplicate and one representative curve is shown (S.D. <10%). **B.** Molecular model of Aβ5-16 interacting with two GM1 molecules arranged into a chalice-like receptacle. **C.** Effects of 10 mM ZnCl_2_ (open squares) and 10 mM NaCl (full squares) on the kinetics of interaction of α-syn34-45/HH peptide with GM1 monolayers.

The amino acid sequences of the minimal glycolipid-binding domain (GBD) of α-synuclein and Aβ are indicated in Figure 1. Of particular interest is the presence of a pair of histidine residues at position 13 and 14 of Aβ5-16, whereas at the same positions the GBD of α-synuclein contains a serine (Ser-42) and a lysine (Lys-43). Interestingly, an independent phage display-based approach allowed Matsubara et al. to select short linear peptides (e.g. the 15-mer shown in Figure 1), that also bind GM1 with high affinity [29], [30]. It is remarkable that all the GM1-binding peptides selected through this phage display strategy were devoid of histidine [30]. Instead, binding to GM1 involved in these cases a pair of non-contiguous Lys or Arg residues (Figure 1). Although Lys and Arg residues were also involved in the binding of Aβ5-16 to GM1, we identified His-13 and His-14 as the most critical residues. Indeed, we have previously reported that a double mutant of the Aβ5-16 peptide, in which both His-13 and His-14 were replaced by Ala residues, had totally lost its capacity to interact with GM1 [13]. However, it remained to determine which of His-13 and His-14 are actually involved in GM1 recognition or if both residues are required. To this end, we prepared a series of single and double mutants of Aβ5-16 and analyzed their interaction with GM1. In these experiments, a monolayer of ganglioside GM1 was prepared at the air-water interface and the peptide was injected in the aqueous subphase. The interaction of the peptide with the ganglioside was evidenced by an increase in the surface pressure of the monolayer, which was followed in real-time with a platinum probe (see [26] and [31] for a full description of the technique and its application to amyloid proteins). For the wild-type Aβ5-16 peptide, the surface pressure π increased immediately after the injection of the peptide (initial velocity v_i_ = 0.25 mN.m^-1^.min^-1^), and then continued to increase until reaching a maximal value after 30 minutes of incubation. In contrast, the double mutant His-13Ala/His-14Ala did not interact with GM1 (Figure 2A), in full agreement with our previous results [13]. Then single mutants were assayed with the aim to identify which His residue is actually critical for GM1 recognition. Surprisingly, both appeared to be involved to the same extent, since a total loss of interaction was observed for each single mutant (His-13Ala and His-14Ala). Molecular modeling simulations shed some light on this result (Figure 2B). The formation of a stable complex between Aβ5-16 and GM1 required two GM1 molecules forming a chalice-like receptacle for the peptide. Previous studies suggested that such ganglioside dimers are likely to occur in lipid raft domains and that they may play a critical role in the membrane insertion of amyloid proteins [14]. In the trimolecular GM1/Aβ5-16/GM1 complex, His-13 interacted with one GM1, and His-14 with the second one. The total energy of interaction of this complex was estimated to -88.4 kJ.mol^-1^. Interestingly, the binding of His-13 and His-14 to the glycone parts of the GM1 dimer accounted for 30.7% (-25.2 kJ.mol^-1^) of this energy. Because Zn^2+^ ions strongly interact with the imidazole group of histidine and inhibit Aβ oligomerization [32], we analyzed the effect of these cations on the binding of Aβ5-16 to GM1. In this case, the interaction between Aβ-5-16 and GM1 monolayers was studied in presence of zinc chloride in the aqueous subphase. The lack of significant surface pressure increase following the addition of Aβ5-16 underneath the GM1 monolayer indicated that Zn^2+^ ions efficiently inhibited the interaction (Figure 2C). To assess that the effect of Zn^2+^ was not due to a non specific salt effect, the same experiment was performed in presence of NaCl instead of ZnCl_2_ (Figure 2C). In this case, the interaction of Aβ5-16 with GM1 was not significantly affected by the salt (compare this curve with the kinetics of Figure 2A for the wild-type Aβ5-16 peptide incubated with GM1 in absence of salt). Thus these experimental data supported the notion that histidine residues are critical for the interaction between Aβ5-16 and GM1.

### Design of a chimeric peptide derived from the GBD of α-synuclein and Aβ

The modeling data shown in Figure 2B suggested that the cooperation between both GM1 molecules allowed an optimal interaction with the Aβ5-16 peptide driven by histidine residues. Conversely, because it is devoid of these histidine residues, the GBD of α-synuclein (α-syn34-45) does not interact very well with GM1, and so it has a marked preference for GM3 [13]. Given the critical role of histidine for the high affinity interaction of Aβ5-16 with the GM1 dimer, we decided to introduce a similar pair of adjacent His residues in α-syn34-45 and we synthesized a chimeric α-syn34-45/Ser-42His/Lys-43His (Figure 1). This peptide is herein referred to as α-syn/HH.

### Interaction of the chimeric α-syn/HH peptide with GM3 and GM1

**Figure 3:**
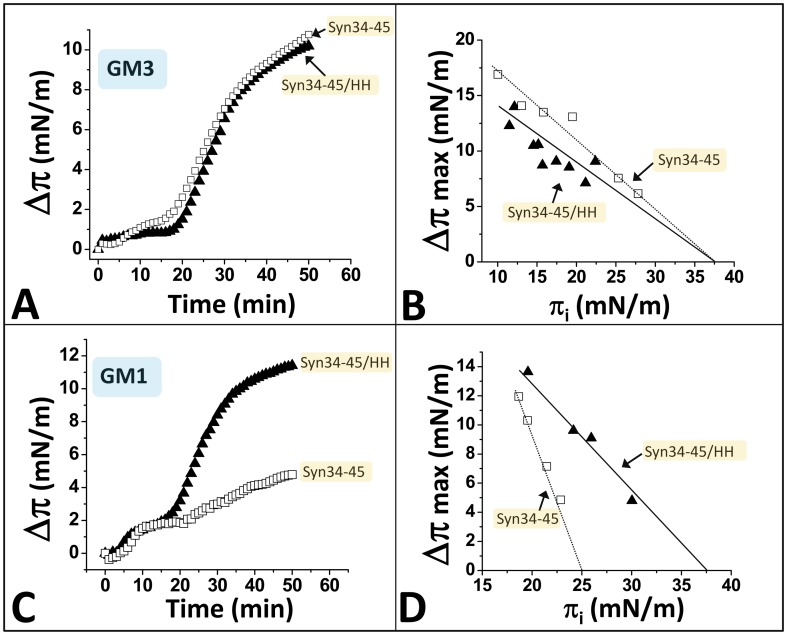
The introduction of His residues within the SBD of α-syn does not alter GM3 recognition and increases its affinity for GM1. *Left panels*. Kinetics of interaction of wild-type α-syn34-45 (open squares) and chimeric α-syn34-45/HH (full triangles) with a monolayer of GM3 (**A**) or GM1 (**C**). In each case the monolayer was prepared at an initial surface pressure of 17.5 mN.m^-1^. All experiments were performed in triplicate and one representative curve is shown (S.D. <15%). ***Right panels.*** Interaction of wild-type α-syn34-45 (open squares) and chimeric α-syn34-45/HH (full triangles) with GM3 (**B**) or GM1 monolayers (**D**) prepared at various values of the initial surface pressure. The maximal surface pressure increase (Δπ_max_) was determined after reaching the equilibrium. The critical pressure of insertion is indicated by the intercept of the slopes with the x-axis.

Since neither Ser-42 nor Lys-43 appeared to be involved in GM3 binding [13], we surmised that this double mutation would not interfere with the interaction of the chimeric α-syn/HH peptide with GM3. As shown in Figure 3, the α-syn/HH peptide behaved exactly as predicted. Its kinetic of interaction with a GM3 monolayer was exactly the same as the wild-type α-syn34-45 peptide (Figure 3A) and the critical pressure of insertion (π_c_) was estimated to 37.5 mN.m^-1^ for both the wild-type and chimeric peptides (Figure 3B). The value of π_c_ is proportional to the binding affinity: this parameter corresponds to the surface pressure of the monolayer above which no interaction occurs because the glycolipids are too densely packed [26], [31]. Thus, the introduction of the pair of His residues in α-syn34-45 did not interfere at all with the GM3-binding capability of the peptide. In contrast, the chimeric α-syn/HH peptide has gained a marked increase of affinity for GM1, which was clearly observed in real-time kinetics studies (Figure 3C). Indeed, the chimeric peptide induced an increase of the surface pressure of the GM1 monolayer immediately upon addition (v_i_  = 0.2 mN.m^-1^.min^-1^), whereas the wild-type α-syn34-45 initially induced a decrease of the surface pressure (v_i_  = −0.5 mN.m^-1^.min^-1^). Moreover, the value of π_c_ measured with GM1 monolayers increased from 25 mN.m^-1^ for the wild-type α-syn34-45 peptide to 37.5 mN.m^-1^ in the case of α-syn/HH, which indicates a significant stronger affinity of the chimeric peptide for GM1 (Figure 3D).

### The chimeric α-syn/HH peptide has a higher affinity for GM1 and GM3 than the wild-type Aβ5-16 peptide

**Figure 4:**
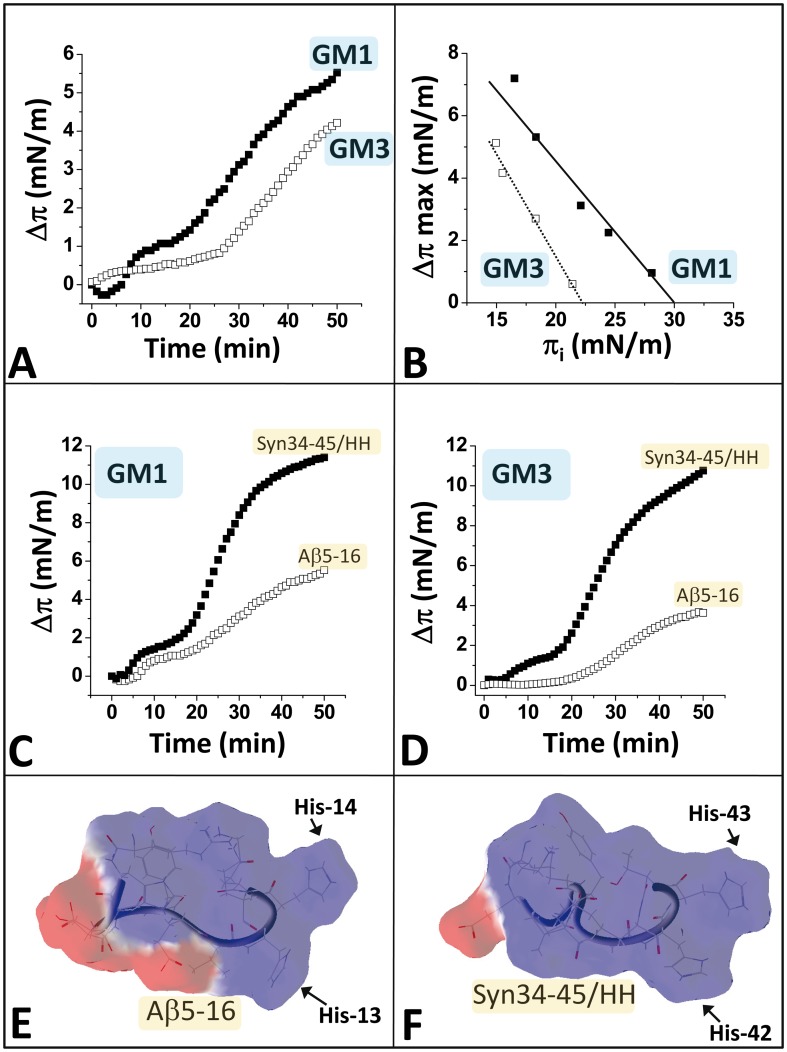
The chimeric peptide has a higher affinity for GM1 and GM3 than the wild-type Aβ5-16 peptide. **A.** Kinetics of interaction of Aβ5-16 with a monolayer of GM1 (full squares) or GM3 (open squares) prepared at an initial surface pressure of 17.5 mN.m^-1^. All experiments were performed in triplicate and one representative curve is shown (S.D. <15%). **B.** Interaction of Aβ5-16 with GM1 (full squares) or GM3 monolayers (open squares) prepared at various values of the initial surface pressure. **C.** Kinetics of interaction of Aβ5-16 (open squares) and chimeric α-syn34-45/HH (full squares) with a monolayer of GM1. **D.** Kinetics of interaction of Aβ5-16 (open squares) and chimeric α-syn34-45/HH (full squares) with a monolayer of GM3. Panels **E** and **F** show the distribution of electric charges on the surface of Aβ5-16 and chimeric α-syn34-45/HH (positive charges blue, negative charges red).

Then we compared the ganglioside-binding properties of the chimeric peptide with those of wild-type Aβ5-16. On the basis of kinetics experiments (Figure 4A), one can see that Aβ5-16 interacted with both GM1 and GM3 monolayers, with a slight preference for GM1. Nevertheless, the critical pressure of insertion π_c_ of Aβ5-16 was 30 mN.m^-1^ for GM1 but only 22.5 mN.m^-1^ for GM3 (Figure 4B). This indicated that Aβ5-16 has a higher affinity for GM1 than for GM3 and that only the interaction with GM1, with a π_c_  = 30 mN.m^-1^ (i.e. the same value as a plasma membrane) is likely to occur in vivo [26]. In all cases, these values were lower than those obtained with the chimeric α-syn/HH peptide, i.e. π_c_ = 37.5 mN/m^-1^ for both GM1 and GM3 (Figure 3B and 3D). A direct comparison of the kinetics of interaction of Aβ5-16 and α-syn/HH with these gangliosides is presented in Figure 4C and 4D. These data showed that whatever the ganglioside tested (GM1 or GM3), the chimeric peptide is more efficient than the wild-type Aβ5-16 peptide (faster initial velocity of the binding reaction and higher surface pressure increase at the equilibrium). Taken together, these data demonstrated that the chimeric peptide has a higher affinity for GM1 and GM3 than the wild-type Aβ5-16 peptide.

### Structural analysis of Aβ5-16, α-syn34-45, and α-syn/HH peptides

**Figure 5:**
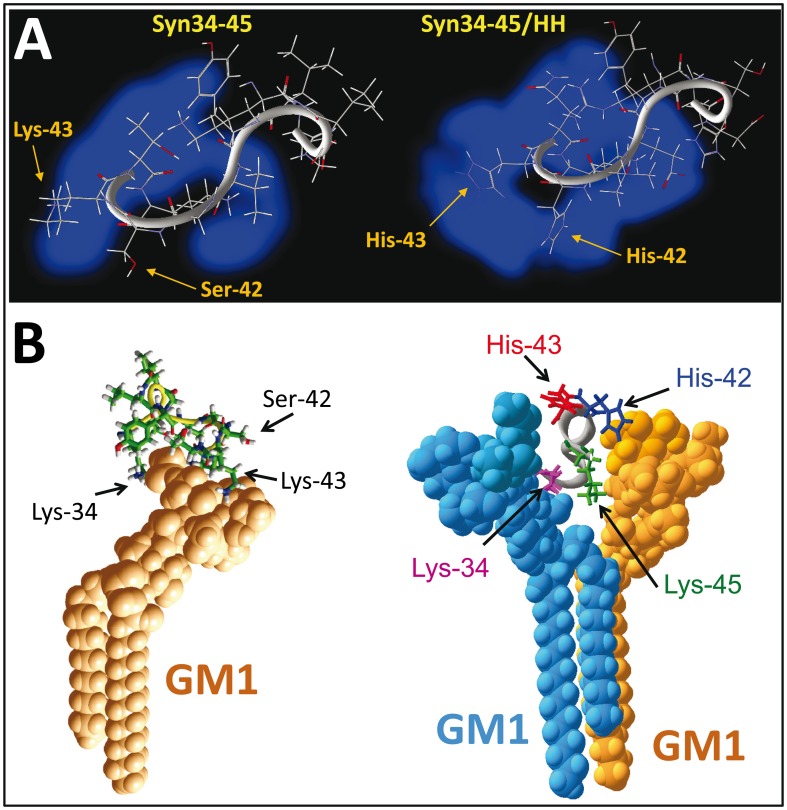
Molecular modeling of the wild-type and chimeric α-syn34-45 peptides. **A.** Visualization of the positive electrostatic potential surface (in blue) of the wild-type α-syn34-45 (left panel) or chimeric α-syn34-45/HH (right panel) peptides. **B.** Molecular modeling simulations of the wild-type α-syn34-45 (left panel) or chimeric α-syn34-45/HH (right panel) peptides interacting respectively with a monomer or a dimer of ganglioside GM1.

**Figure 6:**
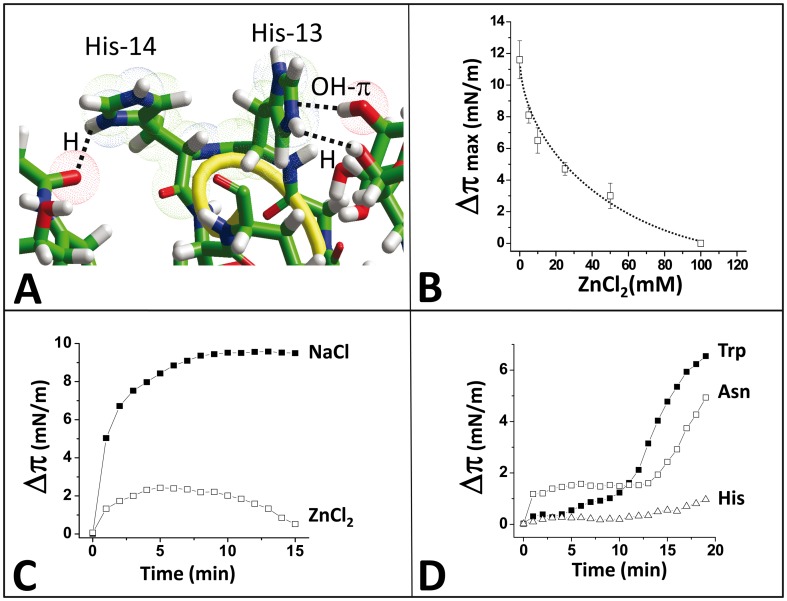
Critical role of His residues in the interaction of the chimeric α-syn34-45/HH peptide and a chalice-shaped dimer of GM1. **A.** Molecular dynamics simulations indicated that the imidazole group of His-14 can form a hydrogen bond with the oxygen atom of the N-acetyl group of the sialic acid of one GM1 molecule (distance 2.5 Å). His-13 interacts with the terminal galactose residue of the second GM1 molecule through a combination of a hydrogen bond (His-13 donor group, oxygen of the OH group of the sugar being acceptor) and an OH-π bond. This coordinated network of interactions involving His-13 and His-14 allowed an efficient connection of the chimeric peptide to both GM1 molecules. In all cases the sialic acid residue of each GM1 ganglioside plays a critical role in the interaction, either directly for His-14 or by allowing the terminal galactose residue to be at a close distance of the imidazole ring of His-13 (2.6 Å for the OH-π bond). The experimental validation of the molecular modeling studies of α-syn34-45/HH-GM1 complex is detailed in the other panels. **B.** Dose-dependent effect of ZnCl_2_ on α-syn34-45/HH interaction with GM1 monolayers. Results are expressed as mean ± SD (n = 4). **C.** Effects of ZnCl_2_ (open squares) and NaCl (full squares) on the kinetics of interaction of α-syn34-45/HH peptide with GM1 monolayers. **D.** Effects of Trp (full squares), Asn (open squares) and His (open triangles) on the kinetics of interaction of α-syn34-45/HH peptide with GM1 monolayers. In these cases the peptide concentration was 10 µM, and the aminoacids were at 1 mM.

A structural analysis of Aβ5-16 (Figure 4E) and α-syn/HH (Figure 4F) confirmed that despite the presence of a common pair of adjacent histidine residues, each peptide has a specific shape and a unique distribution of electric charges. In particular, α-syn/HH has an expanded zone of positive charges and a very small negative area compared with Aβ5-16. This may facilitate the interaction with negatively charged lipids such as GM1 and GM3. Indeed, the introduction of the pair of His residues in the α-syn34-45 framework induced a more symmetrical distribution of the electrostatic potential that is clearly visible when the wild-type α-syn34-45 and chimeric α-syn/HH peptides are compared (Figure 5A). As a consequence, α-syn34-45 and α-syn/HH peptides greatly differed in the way they interacted with the anionic glycone headgroup of GM1. As shown in Figure 5B (left panel), α-syn34-45 adopted a curved shape around the protruding sugar part of a monomer of GM1. This allowed the cationic ε-NH_3_^+^ groups of the terminal Lys-34, and Lys-43 to ‘clamp’ the negatively charged glycone domain of GM1. Overall, these electrostatic interactions accounted for 68.8% of the total energy of interaction of the GM1/α-syn34-45 complex (i.e. -27.4 kJ.mol^-1^ of a total energy of -39.8 kJ.mol^-1^). A similar ‘clamp’ topology of a GM1 monomer bound to a synthetic peptide selected by phage display has been previously reported [30]. Because of its more balanced distribution of the electrostatic field, the chimeric peptide could form a stable complex with a dimer of GM1 molecules arranged in a typical chalice-like receptacle (Figure 5B, right panel). As for the GM1/Aβ5-16/GM1 complex, each of the His residues of α-syn/HH interacted with its own GM1 ganglioside, in a way that recalls the wings of a butterfly on the chalice of a flower. A detailed description of the molecular interactions between α-syn/HH and the GM1 dimer, emphasizing the specific contribution of histidine residues, is given in Figure 6A. In this case, the energy of interaction reached -91.7 kJ.mol^-1^, and the histidine residues His-13 and His-14 contributed for as much as 41% of this energy (Table 1). Compared with the wild-type α-syn34-45 bound to monomeric GM1 (-39.8 kJ.mol^-1^), this represents a 2.3-fold increase, which is fully consistent with the involvement of a second GM1 molecule for the binding of the chimeric α-syn/HH peptide. Overall, these data strongly support the notion that His residues are critical for recruiting the GM1 molecules into a functional chalice-shaped dimer able to accommodate the glycolipid-binding domain of amyloid proteins. Several experiments were thus conducted to demonstrate the prominent role of histidine in this mechanism.

**Table 1:** Energetics of interaction between the chimeric α-syn34-45/HH peptide and gangliosides.

Amino acid residue	GM1	GM2	GM3	GM4	GD1a	GD1b	GD3	GT1b
**Lys-5**	−18.1	-	−23.4	-	−17.2	−34.3	−21.1	−24.1
**Glu-6**	−1.6	-	−4.5	−5.5	−7.9	−2.1	-	−3.2
**Gly-7**	-	−3.2	−4.7	−11.3	-	-	-	−2.8
**Val-8**	-	−13.6	−20.8	−4.2	-8.6	-	-	−0.3
**Leu-9**	-	−1.1	−20.6	−1.4	6.0		-	-
**Tyr-10**	-	−2.7	−19.3	−1.4	-	−3.8	−17.7	−10.5
**Val-11**	−11.2	−0.7	−3.7	−24.3	-	-	-	−22.2
**Gly-12**	−2.3	−3.4	−0.4	−5.4	-	-	-	−3.2
**His-13**	−19.0	−26.5	-	−24.9	−8.9	−15.2	-13.7	−16.2
**His-14**	−18.6	−12.7	−11.7	−11.4	−18.8	−17.4	−24.9	−13.5
**Thr-15**	−6.4	−0.4	−3.8	-	−11.8	−1.9	−7.3	−7.0
**Lys-16**	−14.5	-	−20.1	−4.1	−19.6	−12.8	−16.1	−15.8
***TOTAL***	**−** ***91.7***	**−** ***64.3***	**−** ***133.0***	**−** ***93.9***	**−** ***98.8***	**−** ***87.5***	**−** ***100.8***	**−** ***118.8***
***% His***	***41.0***	***61.0***	***8.8***	***38.7***	***28.0***	***37.3***	***38.3***	***25.0***

The energy of interaction (ΔG, in kJ.mol^−1^) has been determined by molecular docking of α-syn34-45/HH on a minimized dimer of each ganglioside, using Hyperchem and Molegro programs. The contribution of His-13/His-14 to the binding reaction is indicated in the last line of the table. Note that the expected contribution of two residues such as His-13/His-14 to a binding reaction involving equally all residues of a 12-mer peptide is 16.7%.

### The binding of α-syn/HH peptide to GM1 is specifically inhibited by zinc and free histidine

First we analyzed the effect of Zn^2+^ cations, which bind to imidazole [32], and interfere with histidine-driven binding reactions (Figure 2C). To this end, the interaction between the chimeric α-syn/HH peptide and GM1 monolayers was studied in presence of various concentrations of zinc chloride in the aqueous subphase. As shown in Figure 6B, Zn^2+^ ions induced a dose-dependent inhibition of the binding reaction, with a half-maximal effect at 20 mM of zinc chloride. Moreover, a total inhibition of the interaction was observed at 100 mM of zinc chloride (Figure 6C). When ZnCl_2_ was replaced by NaCl (100 mM), the interaction between the chimeric α-syn/HH peptide and GM1 monolayers occurred immediately after the injection of the peptide, at a rapid rate (v_i_  = 4.9 mN.m^−1^.min^−1^), and a stable plateau was reached after 10 min of incubation (Figure 6C). This demonstrated that the inhibitory effect of zinc is highly specific.

The involvement of histidine residues in GM1 binding was further assessed by analyzing the interaction between the chimeric α-syn/HH peptide and GM1 monolayers in presence of free histidine as competitor (Figure 6D). In this case, the interaction was significantly delayed and dramatically decreased. In contrast, neither tryptophan nor asparagine could significantly alter the kinetics of interaction of the chimeric peptide with GM1 (Figure 6D). Indeed, after 25 minutes of incubation, the surface pressure of the GM1 monolayers has increased by 8.7 mN.m^−1^ in presence of tryptophan, 7.4 mN.m^−1^ in presence of asparagine, and only 2.1 mN.m^−1^ in presence of histidine (all free amino acids were used at 1 mM, corresponding to a 100-fold excess vs. the chimeric peptide in these experiments). In absence of competing peptide, the surface pressure of the GM1 monolayer probed by α-syn/HH alone was 7.0 mN.m^-1^ after 25 minutes of incubation (Figure 3C). Therefore, these data indicated that among a series of free amino acids with either a nitrogen atom in the side chain (e.g. asparagine) or/and an aromatic structure (tryptophan and histidine), only histidine could competitively inhibit the interaction of the chimeric α-syn/HH peptide with GM1. Together with the specific and dose-dependent inhibitory effect of zinc (Figure 6B), these data confirmed that the histidine residues introduced in the frame of α-syn34-45 do play a critical role in GM1 recognition.

### Cholesterol accelerates the binding of α-syn/HH peptide to GM1

**Figure 7:**
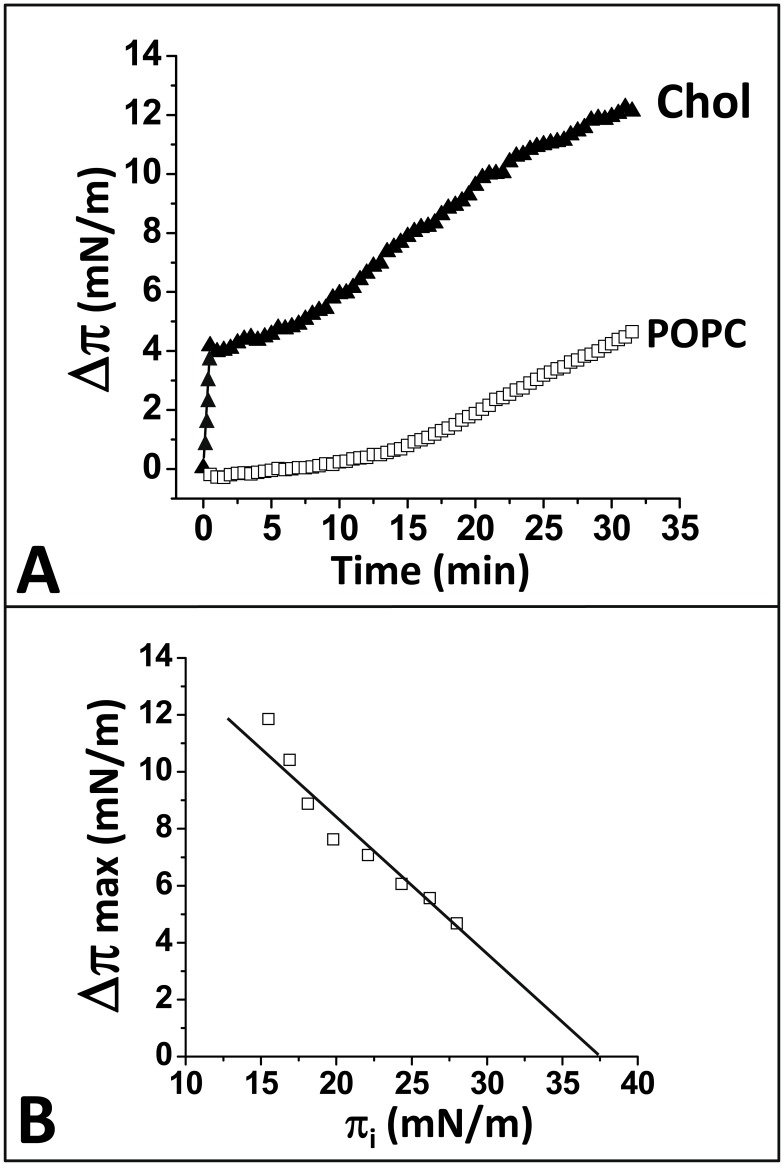
Effect of membrane lipids on the interaction between α-syn34-45/HH and GM1. **A.** Interaction of α-syn34-45/HH with mixed equimolar monolayers of GM1:cholesterol (full triangles) or GM1:phosphatidylcholine (open squares). Chol: cholesterol; POPC, phosphatidylcholine. **B.** Determination of the critical pressure of insertion of α-syn34-45/HH for GM1 monolayers. GM1 monolayers were prepared at various values of the initial surface pressure π_i_ and probed with the chimeric peptide added in the aqueous subphase. The maximal surface pressure increase **Δ**π_max_ was recorded at the equilibrium. The critical pressure of insertion π_c_ (37.5 mN.m^-1^) is determined as the intercept of the linear regression slope with the x-axis.

Then we analyzed the impact of the membrane lipid environment, especially phosphatidylcholine and cholesterol, on the interaction between GM1 and the chimeric α-syn/HH peptide. In these experiments, we prepared mixed monolayers of GM1/cholesterol and GM1/phosphatidylcholine (palmitoyl-oleoyl-phosphatidylcholine, POPC) and followed the kinetics of interaction of α-syn/HH with these monolayers. As shown in Figure 7A, cholesterol considerably accelerated the interaction of α-syn/HH peptide with GM1 (v_i_  = 8.4 mN.m^−1^.min^−1^ to be compared with 0.2 mN.m^−1^.min^−1^ in the case of pure GM1), whereas phosphatidylcholine (POPC) rather tended to slow down the reaction (lag of 10 minutes before the surface pressure begins to increase). This is in line with the well-known effect of cholesterol to form condensed complexes with GM1 [33], allowing a sterol control of glycolipid conformation [34]–[36]. Indeed, similar kinetic curves were obtained when Aβ5-16 was injected underneath GM1/cholesterol and GM1/POPC monolayers (see Figure 5 in ref. [36]). Moreover, the interaction of cholesterol with GM1 has been shown to stabilize the chalice-like conformation of GM1 dimers [36]. In this respect, cholesterol is expected to speed up the interaction without increasing the affinity of α-syn/HH for GM1 (the active conformation of GM1 dimers can be achieved without cholesterol, but with a delayed kinetics, as shown in Figure 7A). The experimental determination of π_c_ for mixed GM1/cholesterol monolayers (37.5 mN.m^−1^, i.e. exactly the value found for pure GM1 monolayers) strongly supports this view (Figure 7B).

### α-syn/HH is a universal ganglioside-binding peptide

**Figure 8:**
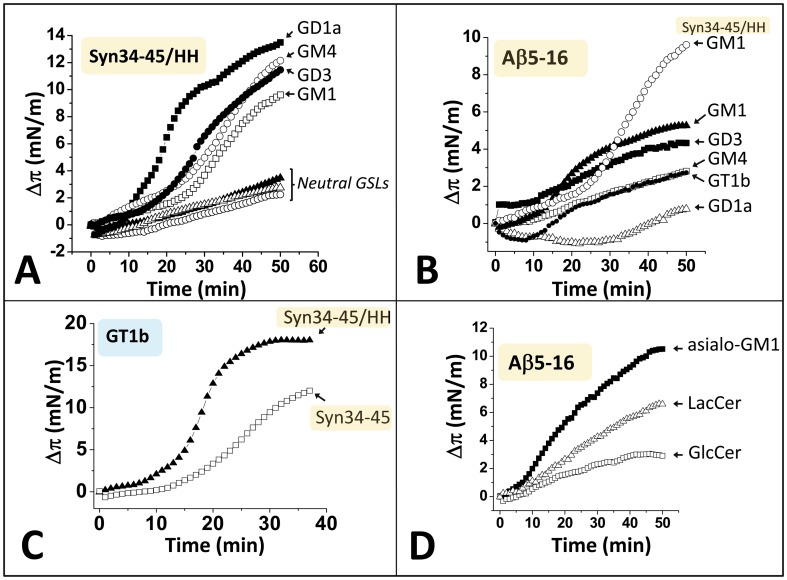
Interaction of the chimeric α-syn34-45/HH and the wild-type Aβ5-16 with gangliosides and neutral glycolipids. **A**. Interaction of the chimeric α-syn34-45/HH peptide with gangliosides: GM1 (open squares), GM4 (open circles), GD1a (full squares), GD3 (full circles), and neutral glycosphingolipids (GSLs): asialo-GM1 (full triangles), LacCer (open circles) and GlcCer (open triangles) in the monolayer assay (the experimental conditions were the same as those of Figure 3A). **B.** Interaction of the wild-type Aβ5-16 peptide with GM1 (full triangles), GM4 (open squares), GD1a (open triangles), GD3 (full squares), and GT1b (full circles). For comparison, the interaction of the chimeric α-syn34-45/HH peptide with GM1 is shown in the same graph (upper curve, open circles). **C.** Interaction of the wild-type α-syn34-45 (open squares) and chimeric α-syn34-45/HH (full triangles) with ganglioside GT1b. **D.** Interaction of the wild-type Aβ5-16 peptide with neutral glycosphingolipids: asialo-GM1 (full squares), LacCer (open triangles) and GlcCer (open squares).

Because the chimeric α-syn/HH peptide combines the most important structural features of α-synuclein and Aβ that ensure proper binding to gangliosides (Lys/Arg at both ends, central Tyr, and His pair), we wondered whether it could interact with all the gangliosides recognized by both amyloid proteins. Thus we analyzed the interaction of the α-syn/HH peptide with a series of glycolipids, including the main gangliosides species expressed in brain and neutral glycolipids as controls. In parallel, molecular dynamics studies were conducted to evaluate the energy of interaction of the chimeric peptide for each chalice-shape dimer of ganglioside. These data are summarized in Figure 8 and Table 1. The results showed that the chimeric peptide displayed a selective affinity for gangliosides (GM1, GM2, GM3, GM4, GD1a, GD1b, GD3 and GT1b), and reacted very poorly with neutral glycolipids (GlcCer, LacCer, asialo-GM1). This indicates that the presence of at least one sialic in the glycone part of the glycolipid is required for binding, in full agreement with the molecular modeling data (Figure 5). In contrast, the wild-type Aβ5-16 peptide recognized essentially gangliosides GM1 and GD3 but reacted poorly with GM4, GD1a and GT1b (Figure 8B). Moreover, Aβ5-16 had a specific pattern of interaction with GlcCer, LacCer, and asialo-GM1 that differed from the weak reactivity of the chimeric α-syn/HH peptide for these neutral glycolipids (compare Figure 8A and Figure 8D).

Most importantly, we found that the chimeric peptide has a higher affinity for gangliosides than the wild-type α-syn34-45 peptide. This is perfectly illustrated for GM1 (Figure 3C) and GT1b (Figure 8C). The only exception to this rule is GM3, which displays the same affinity for both wild-type and chimeric peptides (Figure 3A). Correspondingly, GM3 is the only ganglioside for which the His-13/His14 pair does not play a significant role for the binding of the chimeric peptide (Table 1).

### α-syn/HH inhibits the binding of full-length Aβ1-42 to neural cells

**Figure 9:**
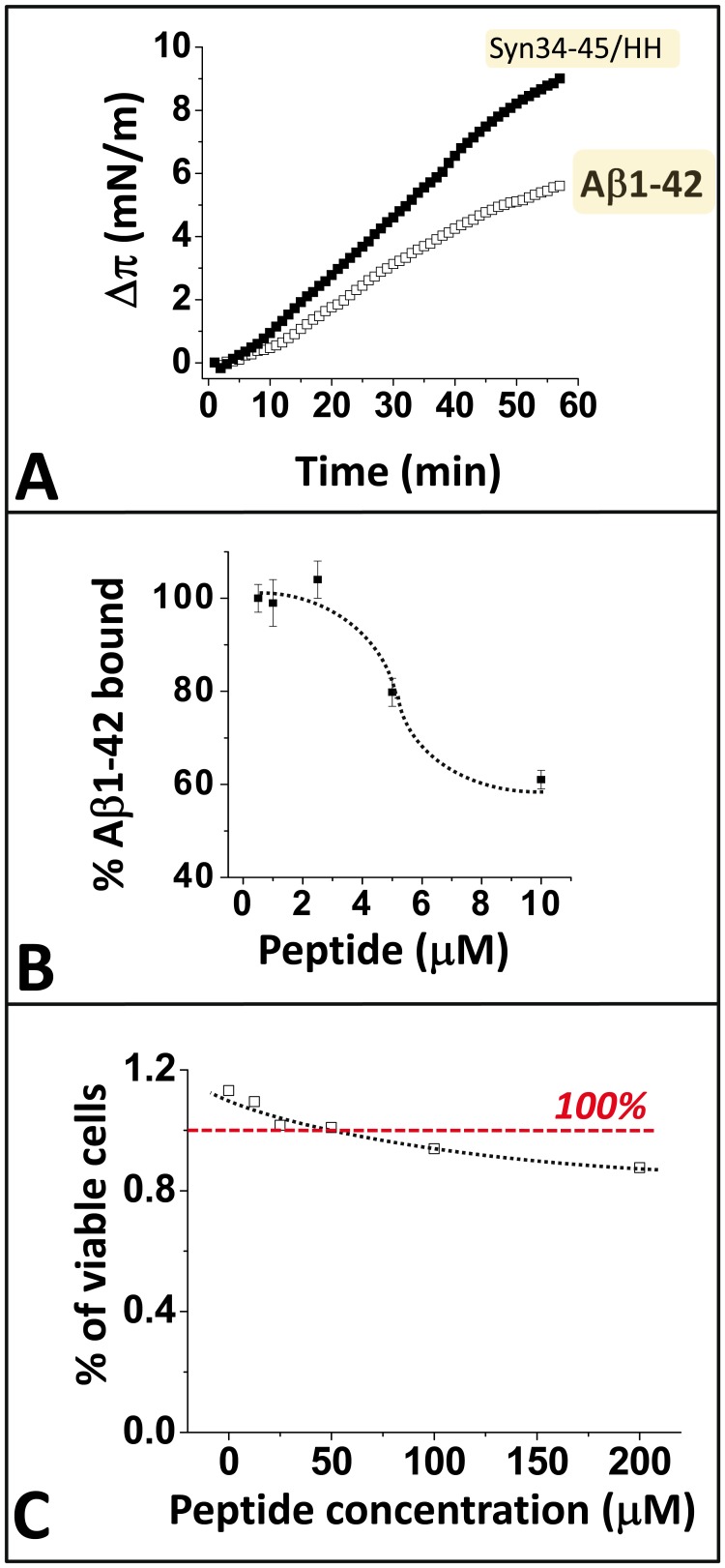
The chimeric α-syn34-45/HH inhibits the binding of Aβ1-42 peptide to neural cells. **A.** Interaction of Aβ1-42 (open squares) and α-syn34-45/HH (full squares) with monolayers of reconstituted raft-like membranes (GM1:Chol: POPC; 2∶1∶1, mol:mol:mol). **B.** Dose-dependent inhibition of biotin-labeled Aβ1-42 binding to SH-SY5Y cells by the chimeric α-syn34-45/HH peptide (sigmoidal fit). The biotin-labeled Aβ1-42 probe and the chimeric peptide were added at the same time onto the cells and incubated for 30 min after which streptavidin-peroxidase was added to the wells. The data are expressed as the percentage of Aβ1-42 specifically bound to the cells as determined by the absorbance at 492nm. Results are expressed as mean ± SD (n = 4). **C.** Evaluation of the cellular toxicity of the chimeric α-syn34-45/HH peptide. The cells (SH-SY5Y) were incubated for 24hr with the indicated concentrations of chimeric peptide in serum-free medium. At the end of the incubation, cell viability was determined with the MTS assay. Results are expressed as mean ± SD (n = 4). The red dotted line indicates a cell viability of 100%.

Finally, we tested the capacity of the chimeric peptide to interfere with the binding of the full-length Aβ1-42 peptide on the surface of neural cells. First we checked that Aβ1-42 recognized reconstituted raft-like membranes containing GM1 and cholesterol in a POPC matrix. As shown in Figure 9A, Aβ1-42 readily interacted with monolayers of these GM1/cholesterol raft-like membranes. As expected, the chimeric peptide also interacted with these membranes, in full agreement with the data of Figure 7B. The binding of Aβ1-42 to the human neuroblastoma cell line SH-SY5Y was analyzed with a spectrophometric assay using a biotin-labeled Aβ1-42 peptide. Our data showed that the chimeric α-syn34-45/HH peptide, added in competition, induced a dose-dependent inhibition of Aβ1-42 binding to the surface of SH-SY5Y cells. At the highest dose tested (10 µM of chimeric peptide), no toxicity of the peptide could be evidenced by the MTS assay (Figure 9C). Therefore, the inhibitory effect of α-syn34-45/HH on Aβ1-42 binding could not be artifactually attributed to a non-specific toxic effect of the peptide.

## Discussion

Our knowledge of glycolipid-protein interactions has rapidly grown since the discovery in 2002 of a common structural domain in sphingolipid-binding proteins, referred to as the SBD [3]. A broad range of SBDs have been identified in phylogenetically-distant proteins including microbial [9], [10], insect [7] and human proteins [11], [13], [14]. These discoveries have enabled to draw a photofit of the SBD, which is typically a looped domain with a central aromatic residue and a basic amino acid at each end [1]. Then subtle variations in the amino acid sequence of the SBD may confer distinct glycolipid-binding properties, as illustrated for Aβ and α-synuclein. [13]. This complicates the elucidation of the code that controls glycolipid/protein interactions. Despite this intrinsic difficulty, we took advantage of the fact that many SBD-derived synthetic peptides retained the glycolipid-binding specificity of the full-length proteins from which they originate [5]-[9]. The possibility to assess the interaction of short synthetic peptides with glycolipid-containing artificial membranes considerably accelerated our comprehension of the molecular mechanisms controling glycolipid recognition by proteins. The use of Langmuir monolayers allowed to precisely control the molar ratio of glycolipids in lipid mixtures [31], and, most importantly, to assess the role of cholesterol in protein-glycolipid interactions [27], [34], [36], [37]. In the present report, we have designed a series of 12-mer peptides derived from Aβ and α-synuclein proteins, and analyzed the insertion of these peptides into ganglioside monolayers. This strategy allowed us to deciphering the biochemical code governing the specificity of interaction of these amyloid proteins with distinct plasma membrane gangliosides: GM1 for Aβ and GM3 for α-synuclein.

Several conclusions can be drawn from this study. (**i**) We showed that both Aβ and α-synuclein display a common, structurally-related glycolipid-binding domain (GBD) with little sequence homology. (**ii**) The high affinity of Aβ for ganglioside GM1 is determined by the presence of a pair of histidine residues (His-13 and His-14). (**iii**) The replacement of either His-13, His-14 or both resulted in a loss of interaction with GM1. In agreement with these findings, saturating the imidazole group of His residues by Zn^2+^ cations also inhibited the interaction of Aβ with GM1 (**iv**) This is because each histidine residue interacts with a distinct GM1 molecule, leading to the formation of a trimolecular complex in which two adjacent GM1 gangliosides form a chalice-like receptacle for the Aβ peptide. (**v**) Having deciphering this glycolipid recognition code, we were able to transform the glycolipid-binding domain of α-synuclein into a universal ganglioside-binding peptide. To this end, we replaced amino acids Ser-42 and Lys-43 of the minimal glycolipid-binding domain of α-synuclein (α-syn34-45) by two histidine residues (α-syn/HH). The resulting chimeric α-syn/HH peptide fully retained the ability to recognize ganglioside GM3 and has acquired the capacity to bind to condensed complexes of GM1 (including dimers) at high surface pressures. In fact, the chimeric peptide had a higher affinity for GM1 than the wild-type Aβ5-16 peptide. (**vi**) The active dimeric conformation of GM1 was efficiently stabilized by cholesterol which boosted the binding of the α-syn/HH peptide without affecting its affinity for GM1. (**vii**) In marked contrast with the wild-type Aβ5-16 peptide, the chimeric α-syn/HH peptide interacted almost exclusively with gangliosides, ignoring neutral glycolipids that are devoid of sialic acids (GlcCer, LacCer, asialo-GM1). Neither Aβ5-16 nor α-syn34-45 displayed such a high and specific affinity for gangliosides. For this reason the chimeric α-syn/HH peptide can be considered as a universal ganglioside-binding peptide with a particular affinity for condensed cholesterol/ganglioside complexes found in lipid raft domains of the plasma membrane [2], [4], [33].

Molecular dynamics simulations suggested an interesting explanation for the role of histidine residues in the preferential recognition of chalice-shaped GM1 dimers vs. GM1 monomers. There is a similarity between the wild-type α-syn34-45 GBD peptide and the GM1-binding peptide selected by phage display: both contain a pair of non-consecutive basic residues (Lys) but are devoid of histidine. Due to the presence of several methylene groups, the side chain of Arg and Lys residues is relatively long and highly flexible (Figure 1). In both cases, the aliphatic chain is ended by a positively charged residue that can interact with the negative polar headgroup of GM1. These side chains form a clamp (Figure 1) which grips the sugar part of a single GM1 molecule [30]. In the wild-type Aβ5-16 and in the chimeric α-syn/HH peptide, the vicinal His residues are neither flexible nor long enough to form that kind of grip. Instead, their imidazole rings can be metaphorically compared to the wings of a butterfly gathering the ‘chalice’ of the ganglioside dimer. This particular and functionally active conformation of GM1 gangliosides is controled by cholesterol, which induces a tilt in the orientation of the sugar part with respect to the main axis of GM1 [36]. Consistent with the prominent role of His residues in GM1 recognition, we showed that the interaction of the α-syn/HH peptide with GM1 was specifically and dose-dependently inhibited by Zn^2+^ ions, which bind to the imidazole group of histidine, and by an excess of free histidine (Figure 6). These experimental data fully confirmed our modeling studies.

Considering its high affinity for gangliosides in their natural lipid environment, the chimeric α-syn/HH peptide may have potential therapeutic applications in infectious and neurodegenerative diseases involving gangliosides [2], [4], [12], [18], [20], [38], [39]. It is noteworthy that the pair of histidine residues His-13 and His-14 has been previously identified as a potential target for inhibition of Aβ toxicity [40]. Moreover, ganglioside GM1 is a major target for Aβ1-42 on the surface of brain cells, stimulating its adhesion on lipid rafts, its clustering and the formation of highly neurotoxic oligomers and aggregates [41]-[45]. Recently, Hong et al. have elegantly demonstrated that Aβ oligomer-mediated neurotoxicity (LTP impairment) in mouse hippocampal slices could be inhibited by the B-subunit of cholera toxin, a classic GM1-binding protein [20]. The chimeric α-syn/HH peptide described in the present study binds GM1 with high affinity and it is not toxic. It can interact with a broad range of gangliosides (Table 1) and it inhibits the binding of Aβ1-42 to neural cells. Moreover, plasma membrane gangliosides (including GM1 and GM3), which are recognized by the SBD of HIV-1 surface envelope glycoprotein gp120 [21], [46], have been involved in HIV infection and cell-to-cell transmission [38], [39], [47]. Thus there is an urgent need to develop therapeutic molecules targeting cell surface gangliosides [2], [12]. Future studies will determine whether the chimeric α-syn/HH peptide can be used as a universal inhibitor of the membrane insertion process of various amyloid and pathogen proteins [2], [4], [26], [48], and/or as a potential common amyloid epitope [49], [50] for vaccine approaches against Alzheimer's, Parkinson's and other neurological diseases.
